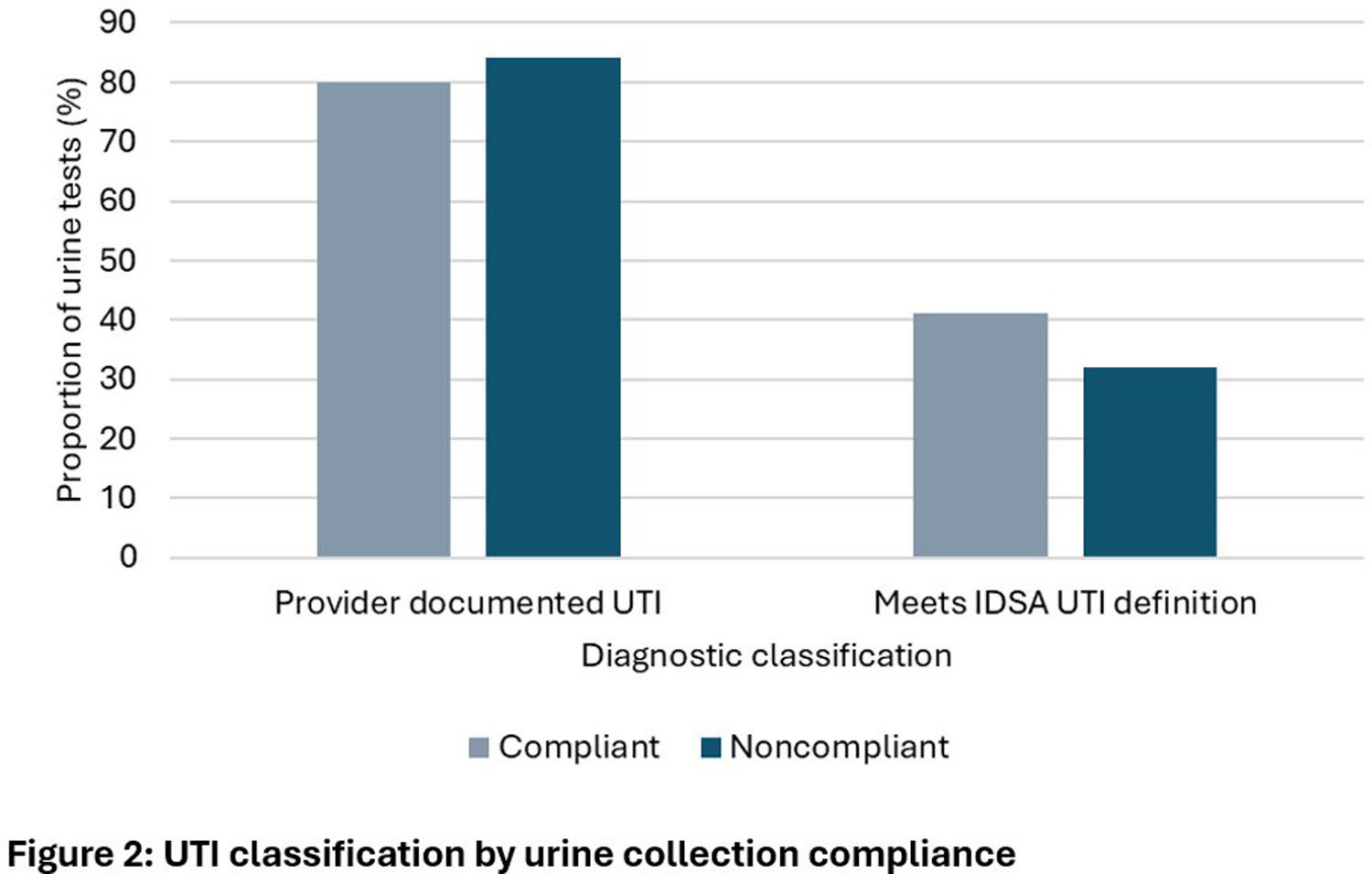# 149 Infection Control Education Needs and Knowledge Gaps among Environmental Services Staff in a Tertiary-Care Hospital

**DOI:** 10.1017/ash.2026.10553

**Published:** 2026-06-23

**Authors:** Joy Abou Farah, Alexia El Khoury, Luke Kabbara, Ethan Martin, Jose Andres Portillo, Zainab Albar, Aoi Yogo, Tina Lewis, Jay Krishnan, Leila Hojat, Elie Saade, University Hospitals Cleveland Medical Center

**Affiliations:** 1 Case Western Reserve University; 2 Case Western Reserve University/ UH Hospitals; 3 University Hospitals, Cleveland; 4 University Hospitals; 5 University Hospitals Health System; 6 University Hospitals, Case Western Reserve University; 7 Emory University Hospital Midtown

## Abstract

**Background:** Urine testing is common among hospitalized patients with indwelling urinary catheters, yet variation in collection practices may compromise diagnostic accuracy and contribute to unnecessary downstream interventions. Guidelines recommend avoiding sampling from indwelling catheters when feasible to reduce contamination and detection of asymptomatic colonization. However, real-world compliance and its relationship to diagnostic urine test results, subsequent UTI diagnosis, and antimicrobial prescribing remain incompletely characterized. **Methods:** We conducted a retrospective medical record review of adult patients who underwent urine testing between March and November 2024 and had an indwelling urinary catheter or catheter removal within the prior 24 hours. Urine samples were classified as compliant if obtained after catheter removal and noncompliant if collected with a catheter in place. Analyses included all urine samples, with urinalysis-specific analyses limited to samples with available results. Urine collection practices, urinalysis parameters, urine culture outcomes, provider-documented UTI diagnoses, concordance with symptom-based criteria and IDSA/NHSN definitions, and antimicrobial prescribing following urine testing (receipt, timing, route, and duration) were summarized descriptively and stratified by compliance status. **Results:** A total of 921 urine samples were included; 663 had an initial urinalysis with reflex urine culture, while the remainder underwent urine culture alone. Samples compliant with recommended collection practices accounted for 59% of cases. Among samples with urinalysis, overall positivity was similar in compliant and noncompliant samples (57% vs 55%), with comparable urinalysis component positivity across compliance groups. Urine culture positivity was also similar across compliance groups in both reflex-culture and culture-only samples (29% vs 27% and 27% vs 29%). Gram-negative organisms predominated (13-15%), followed by fungi (7-12%) and Gram-positive organisms (2-4%), with fungal isolates more common among noncompliant samples. Antimicrobial therapy was prescribed in 19% of compliant samples compared with 15% of noncompliant samples and was frequently initiated before culture results in both groups. Median treatment duration was similar (7 vs 6 days). Provider-documented UTI diagnoses were common (80% vs 84%); however, only a portion met standardized UTI definitions (IDSA criteria: 41% compliant vs 32% noncompliant), with greater concordance observed among compliant samples. **Conclusion:** Recommended urine collection practices were followed in just over half of samples, with similar urine test positivity across compliance groups. Differences in organism distribution, antimicrobial prescribing, and concordance between provider-documented and standardized UTI diagnoses suggest that urine collection practices may influence diagnostic classification and antimicrobial decision-making in hospitalized adults.

**Figure:**
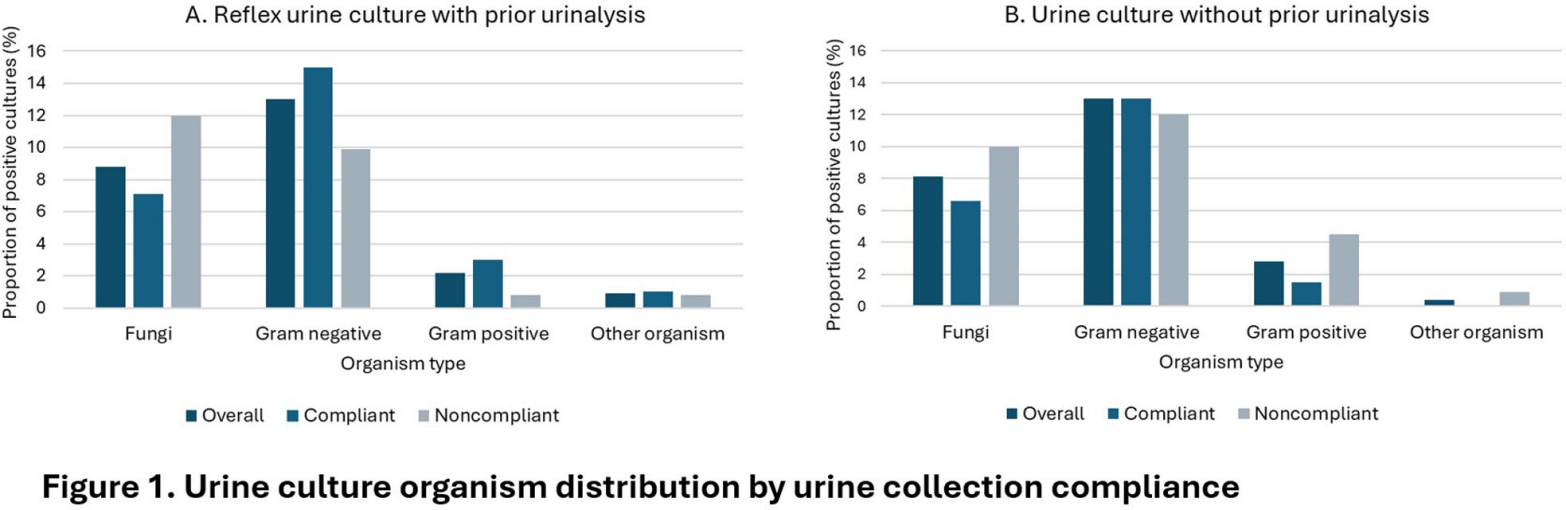

**Figure:**